# Comparative evaluation of microleakage and penetration depth of ACP containing pit and fissure sealant and flowable composite – An *in-vitro* study

**DOI:** 10.4317/jced.61756

**Published:** 2024-08-01

**Authors:** Poorvi Bartaria, Priyanka Sharma, H.P-Suma Sogi, Mansi Jain, Prinka Shahi, Roopam Kapoor

**Affiliations:** 1 Post Graduate. Maharishi Markandeshwar College of Dental Science and Research; 2 Reader. Maharishi Markandeshwar College of Dental Science and Research; 3 Professor and HOD. Maharishi Markandeshwar College of Dental Science and Research; 4 Professor. Maharishi Markandeshwar College of Dental Science and Research; 5 Maharishi Markandeshwar College of Dental Sciences and Research

## Abstract

**Background:**

The present *in vitro* study evaluated and compared the microleakage and penetration depth of ACP containing pit and fissure sealant with flowable composite.

**Material and Methods:**

Sixty extracted non-carious premolars were categorized into four groups of 15 samples each. Sealant was applied after the Occlusal surfaces of the sample teeth were prophylactically treated with pumice slurry. Later, the teeth were thermocycled and immersed in methylene blue for a period of 24 hours. The samples were buccolingually sectioned and analysed under a stereomicroscope. Statistical analysis was carried out to compare the microleakage and depth of penetration.

**Results:**

Flowable nanocomposite gave comparable results with that of the sealant in terms of microleakage. Nano-hybrid flowable composite performed better with respect to depth of penetration between tested materials with the difference being statistically significant.

**Conclusions:**

Flowable nanocomposite can be considered as a promising substitute for sealing fissures and thus can be endorsed to caries susceptible pediatric patients.

** Key words:**Depth of Penetration, Sealants, Microleakage.

## Introduction

Dental caries is the most prevalent dental illness, and it continues to be the most common condition in children despite all preventive measures. The increased susceptibility of occlusal surface to caries is due to the fact that the fissures provide a protected niche for plaque accumulation (1). Two main strategies are used to deal with deep pits and fissures: topical fluoride application and pit and fissure sealants. Topical fluoride application is more effective on smooth surfaces of the tooth, whereas the pit and fissure sealants are used successfully on the occlusal surface (2)

Sealing the surface with sealants creates a physical barrier that blocks the biofilm’s nutrition and, as a result, inhibits biofilm growth (3). Pit and fissure sealants act by the physical obstruction of pits and fissures, which prevents colonization of the pits and fissures with new bacteria (4).

However, the effectiveness of pit and fissure sealants relays on their long-term retention (5).

The intent of the study was to gauge the efficacy of different materials used as pit and fissure sealants, by evaluation of microleakage and their penetration depth into the pits and fissures.

## Material and Methods

A total of 60 posterior non-carious extracted teeth were included in the study. The collected teeth samples once obtained were brushed and washed. Debris and calculus were removed from the surface of teeth with an ultrasonic scaler. Each tooth was treated with pumice slurry using a prophy cup. The samples were then thoroughly washed and dried. Later, the collected teeth samples were placed in normal saline to prevent them from dehydration which could cause them to turn brittle.

60 sample teeth were then divided into groups of four containing 15 teeth.

• Group I ACP containing pit and fissure sealant for microleakage (Prevest PF Seal)

• Group II Flowable Composite for microleakage( Prevest Fusion Flow)

• Group III ACP containing pit and fissure sealant for Depth of Penetration (Prevest PF Seal)

• Group IV Flowable Composite for Depth of Penetration (Prevest Fusion Flow)

-Sealant application

All the samples of Group I and Group III were collected and their occlusal surfaces were etched with Avue Etch (37% Orthophosphoric acid) for an interval of 30 seconds. The etched samples were washed and dried.

The sealant was applied according to the manufacturer’s instructions. The sealant was then placed on the occlusal surface of the teeth. The explorer tip was moved along the fissure to prevent the entrapment of air. Light cure unit was used for 20 seconds on the occlusal surface of the collected teeth for polymerization.

Later, the samples were placed in distilled water after sealant placement.

-Flowable composite application

All the samples of Group I and Group III were collected and their occlusal surfaces were etched with Avue Etch (37% Orthophosphoric acid) for an interval of 30 seconds. The etched samples were washed and dried.

After drying of the etched surfaces, light cure nano-hybrid Flowable Composite (Fusion Flo) was applied with an applicator tip directly to the occlusal surface of teeth. A light cure unit was used and the material was polymerized for 40 seconds (According to the manufacturer’s instructions).

-Thermocycling 

The samples in all four groups were kept in distilled water at ambient temperature after sealant placement. The samples were then thermocycled between 5°C and 55°C with an immersion time of 30 seconds.

-Sticky wax application and dye immersion

All surfaces of the specimen teeth were coated with nail varnish. A 2 mm margin was left around the border of the sealant. After completion of thermocycling, sticky wax was applied to the apex of each tooth sample to prevent any leakage. This was done for all of 60 teeth after which the teeth samples were immersed in 5% methylene blue at 37°C for a time period of 24 hours. Later, the samples were removed from the solution and were rinsed under running water for the removal of excess dye and later dried.

-Sectioning of the samples

The sample teeth were sectioned in a Bucco-lingual direction occlusally using a highspeed straight handpiece with a SS White diamond disc along with water spray.

-Evaluation of microleakage 

Scoring Criteria 6

Score 0 : No dye penetration

Score 1 : Dye penetration restricted to the outer half of the sealant.

Score 2 : Dye penetration to the inner half of the sealant.

Score 3 : Dye penetration into the underlying fissure.

**Figure 1:**
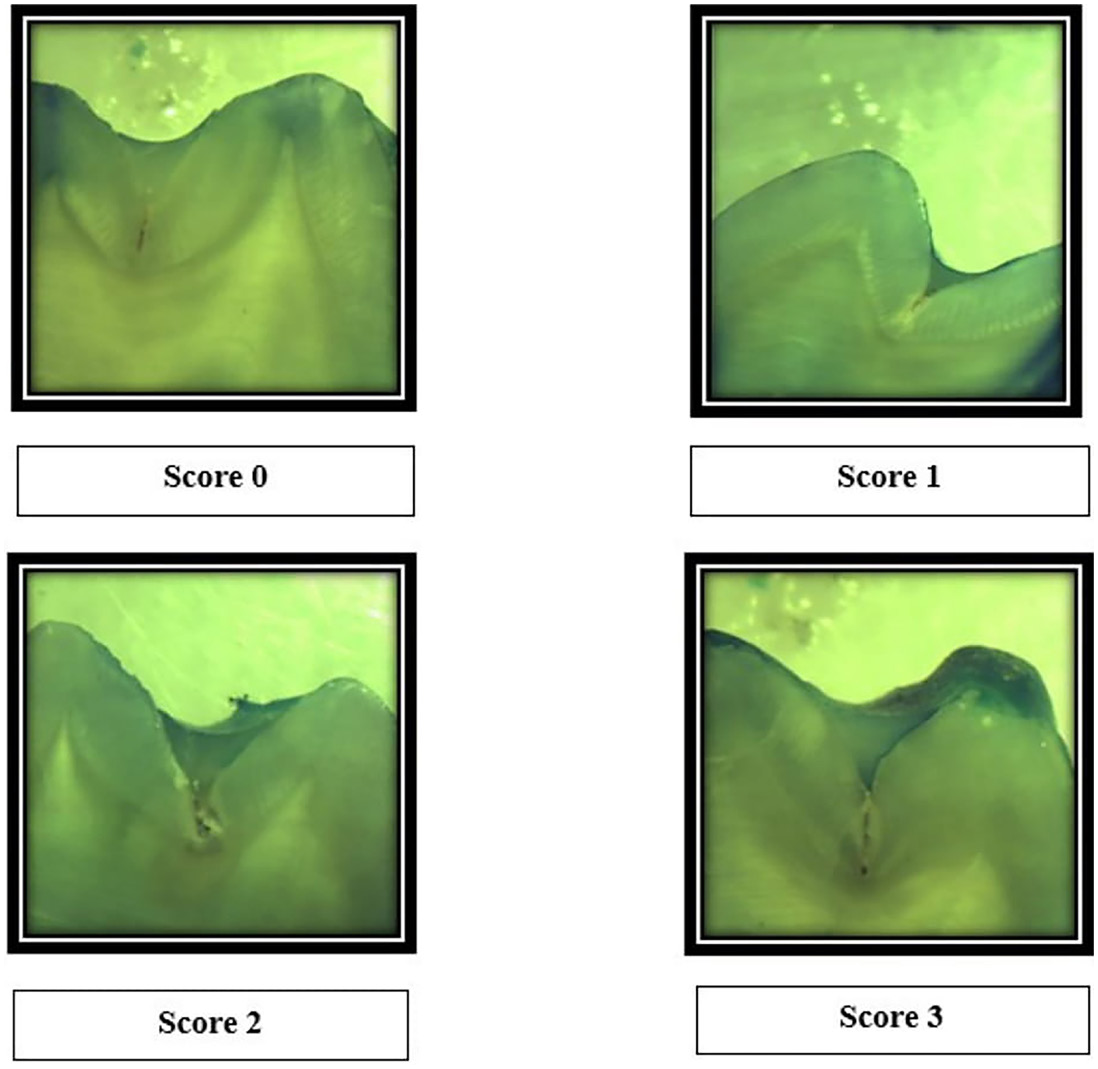
Photographs of samples showing different scores.

**Figure 2:**
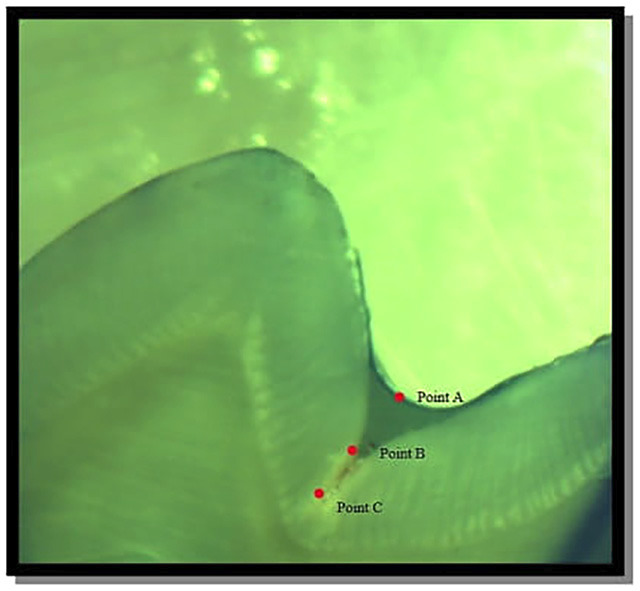
Points of reference to calculate the fissure depth and penetration os sealant.

**Figure 3:**
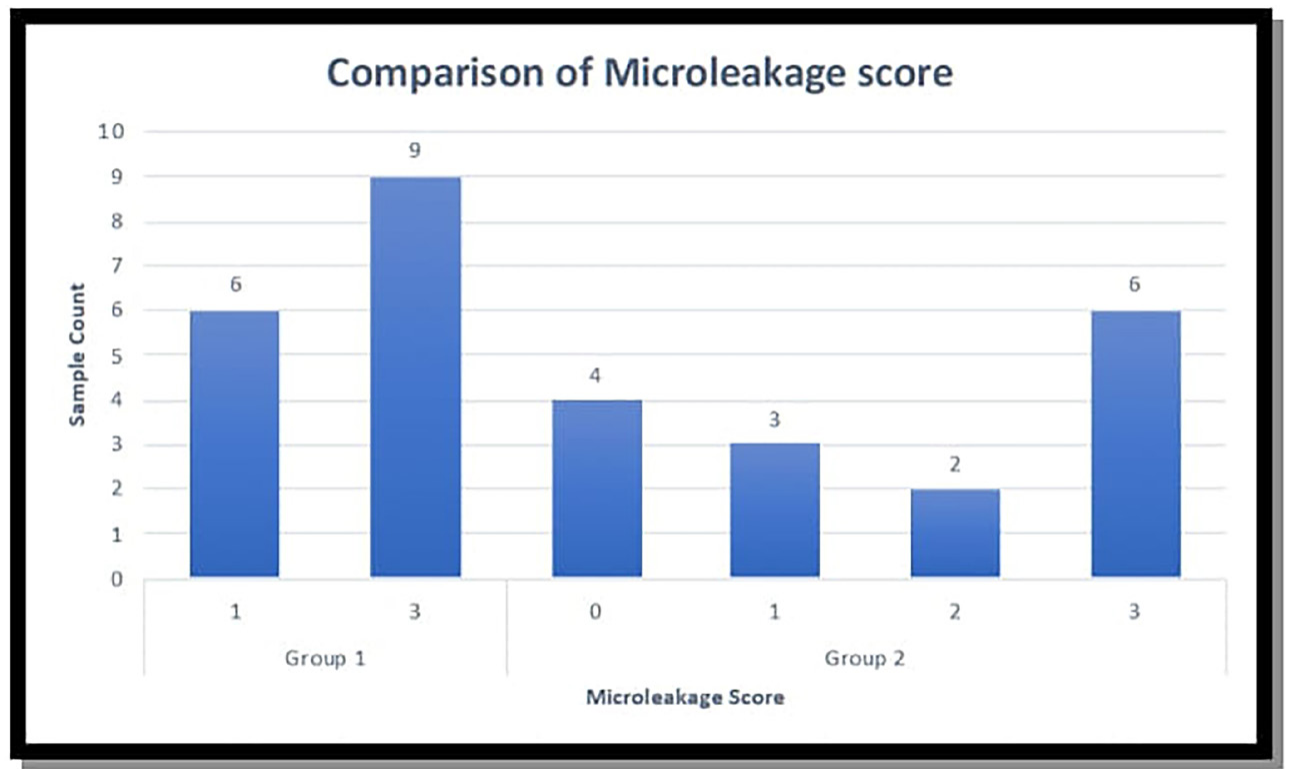
Dye penetration among the two groups.

The samples were assessed and graded by a single examiner using Ovrebo and Raadal criteria for the evaluation of dye penetration.6 The images were captured and analyzed by using TS Capture Software. Measurement tools present in the software were used, (Figs. 1-3).

The recorded values of the present study were tabulated and statistically analyzed using Statistical Package for Social Sciences (SPSS) Software version 21. Paired t-Test was used for the comparison of (two or more groups) in microleakage evaluation group.

Percentage Depth of Penetration was calculated and paired t-test was used for comparative evaluation between the two groups. The sample teeth were examined and depth of penetration was analyzed under a stereomicroscope under 40X magnification.

The samples were assessed by a single examiner using TC Capture Software for the evaluation of depth of penetration.

## Results

-Microleakage

It was evident that within Group 1, there are 9(60%), instances with a microleakage score of 3 and 6(40%) instances with a microleakage score of 1.

Conversely, within Group 2 (Fusion Flo) microleakage score of 0 was seen in 4(26.6%) samples, score 1 was seen in 3(20%) of the samples, score 2 was seen in (13.3%) of the samples and score 3 was seen in 6(40%) of the samples, encompassing three, two, and six instances, respectively.

The *p-value* obtained was 0.84823517 which was substantially larger than the conventional significance level of 0.05.

Therefore, based on the analysis, a statistically significant difference could not be asserted in the mean microleakage values among the material groups.

-Depth of Penetration (DOP)

Group 4(Fusion Flo composite) exhibited greater average DOP when compared to Group 3 (PF seal). The flowable composite group performed better significantly in terms of DOP when compared to PFS. The calculated *p-value* of 0.02170494 was smaller than the commonly used significance level of 0.05.

Thus, a statistically significant difference was observed between the two material groups, ( Table 1).

## Discussion

Pit and fissure caries accounts for about 90% of the caries of permanent posterior teeth in adolescents and 44% in the primary teeth in children (7). The unique morphology of pits and fissure pattern makes them prone to dental caries in permanent dentition (1).

For several decades dentists have recognized that there exist some deep occlusal lesions in posterior teeth, which miss being detected as the occlusal surfaces remained ostensibly intact until large parts of the crown have been destroyed (8).

Pits and fissures are harder to clean because of their plaque-retentive nature, which renders them to be more prone to carious lesions than smooth surfaces and may prevent fluoride therapy from protecting them (9). Pit and fissure sealants function as a physical barrier that isolate pits and fissures from microorganisms and their by-products preventing the build-up of dental plaque (10).

An optimal sealing material should possess biocompatibility, retention, and resistance to wear and abrasion. Retention of the sealants hold their protective barrier, minimizing the risk of caries (11). Fissure sealant therapy’s efficacy to prevent dental cavities is directly influenced by the flowability of the sealants of how well they fill in anatomical flaws, and how well they bind to tooth structural caries (12).

Although dental sealants can be made of a variety of materials, but composite resin is most frequently employed (13). The use of nanotechnology to flowable composites allows the development of a composite that maintained the flowable composite’s elasticity, adaptability, and favourable handling characteristics while minimizing polymerization shrinkage by nearly 20% (14). This composite had the mechanical properties, wear resistance, strength, enhanced polishability, excellent polish retention, and translucency of a conventional resin composite (14).

It has been demonstrated that using various fissure sealants containing amorphous calcium phosphate is effective in preventing enamel demineralization (15). ACP exhibits potential for remineralization and anti-cariogenic qualities.16 Prior research has demonstrated that sealants containing ACP can encourage the remineralization of artificially created carious lesions on smooth enamel surfaces (17).

The goal of the current *in-vitro* investigation was to assess two FS materials and compare them in order to determine the depth of penetration and microleakage.

A thin layer of plaque that is held on the cracks in teeth may compromise the sealant material’s bond with the enamel (18). As a result, an ultrasonic scaler was used to remove debris, calculus, and soft tissue from the tooth surfaces.

The study’s sample teeth were given an aqueous pumice slurry pre-treatment. This was in line with research done by Blackwood *et al*. 2002, which found that the majority of clinicians still regards the use of pumice prophylaxis and the acid-etch procedure as the gold standard for cleaning cracks before sealant application (19).

In the current investigation, the tooth surfaces were etched with 37% phosphoric acid for 30 seconds in order to improve sealant bonding. When the etchant is applied, the tooth surface becomes rougher. This promotes the sealant’s penetration into the enamel and helps it adhere to the tooth surface (20).

The prevalence of recurring caries beneath sealants can be increased by bacteria and their fluids penetrating the sealant tooth margin due to improper marginal seal displayed by sealants (18).

The chemical dye penetration technique is more precise than bacterial penetration because the dye particles have the same size as bacterial endotoxins and a smaller diameter than bacteria (21). Pit and fissure sealants physically obstruct the pits and fissures from the surrounding oral environment by adhering to the acid-etched enamel surface. This serves as the sealant’s preventive role (10).

Similar to a study conducted by Butail A and colleagues, the sample teeth in this investigation underwent thermocycling for 250 rounds in a water bath with the temperature set between 5°C and 55°C (22). In order to simulate the temperature and chronically harsh conditions that the sealants are subjected to, thermocycling was performed (23).

the apices of the sample teeth were covered with sticky wax, and their coronal surfaces were coated in two coats of nail polish before being let to dry. This was carried out in accordance with a comparable study done by Hatirli H *et al*. 2018 (10).

A dye’s penetration may reveal an imperfect seal. In the current investigation, the two investigated sealant groups underwent dye penetration testing to assess microleakage. As recommended by Birkenfeld *et al*., (24) the samples were submerged in methylene blue for a duration of 24 hours and then observed. According to some researchers, methylene blue leaks similarly to butyric acid (25) a metabolic product of microorganisms that penetrates further than Indian ink.

An assessment technique that has been utilized to gauge the internal and marginal adaptability of restorative materials is the microleakage test (26). Thus, once the samples were submerged in 5% methylene blue, microleakage was measured in this investigation.

All of the groups in the current investigation demonstrated dye penetration to a degree that was comparable to the findings observed in line with those published by Butail A *et al*. (27)

The microleakage results obtained in this study were similar to those obtained by Imam S *et al*. (2015). The study depicted that self-adhering flowable composite, exhibited less microleakage when compared with pit and fissure sealant (28).

Retention of the sealant is affected by DOP of a material which is why penetration of the material is an important criterion for the long life of the sealant (10). Regarding the depth of penetration, a noTable distinction was seen between flowable composite and pit and fissure sealant.

In this study, flowable composite and amorphous calcium phosphate-containing fissure sealant were tested for depth of penetration. The results obtained from the present study were in accordance with another study done by Singh S *et al*. 2011 (29) where nanocomposite exhibited greater penetration depth when compared with fissure sealant. The results of a study done by Hatirli H *et al*. 2018 10 were concomitant with the present study where the depth of penetration observed was greater for flowable composites.

The results obtained were consistent with a study conducted by Dixit A *et al*. in 2021 (30) wherein flowable composite demonstrated superior penetration depth and reduced microleakage in comparison to pit and fissure sealants.

## Conclusions

it has become imperative to manage and control the advancement of carious lesions. The necessity of preventive measures in the initial years of life is becoming more and more apparent to dental teams and pediatric healthcare professionals.

Fusion Flo nanohybrid composite is an excellent flowable composite. It has the best surface affinity, flowability, and penetration into even the hardest to reach places, such as pits and fissures. In comparison to amorphous calcium phosphate-containing pit and fissure sealant PF Seal, this indicates a deeper penetration.

Thus, flowable composite could be used as a promising alternative to amorphous calcium phosphate containing pit and fissure sealant in preventing the initiation of occlusal caries.

## Figures and Tables

**Table 1:** Evaluation of Depth of Penetration values among the two groups.

GROUPS	N	MEAN VALUE	MINIMUM	MAXIMUM	VARIANCE
GROUP 3: PF Seal	15	62.4906667	32.4	98.29	474.500992
GROUP 4: Fusion Flo	15	76.396	47.31	99.39	251.830269
Calculated p-value of 0.02170494					

## Data Availability

The datasets used and/or analyzed during the current study are available from the corresponding author.
